# Advances in CAR-T therapy for central nervous system lymphoma

**DOI:** 10.3389/fimmu.2026.1858455

**Published:** 2026-08-21

**Authors:** Xiumei Feng, Jing Yang, Xiaofei Xu, Wen Kai, Yanxin Huang, Shanshan Zhang, Lin Su, Leisheng Zhang

**Affiliations:** 1 Department of Hematology, The Fourth People’s Hospital of Jinan Affiliated to Shandong Second Medical University, Jinan, China; 2 Laboratory of Precision Medicine, The Fourth People’s Hospital of Jinan Affiliated to Shandong Second Medical University, Jinan, China; 3 Department of Neurosurgery, The Fourth People’s Hospital of Jinan Affiliated to Shandong Second Medical University, Jinan, China; 4 Rehabilitation & Health Care Ward, The Fourth People’s Hospital of Jinan Affiliated to Shandong Second Medical University, Jinan, China; 5 Shandong Provincial Key Medical and Health Laboratory of Blood Ecology and Biointelligence, Jinan Key Laboratory of Medical Cell Bioengineering, Science and Technology Innovation Center, The Fourth People’s Hospital of Jinan Affiliated to Shandong Second Medical University, Jinan, China; 6 Department of Respiratory Medicine and Critical Care, The Fourth People’s Hospital of Jinan, Affiliated to Shandong Second Medical University, Jinan, China

**Keywords:** CAR-T therapy, CNS lymphoma, neurotoxicity syndromes, primary central nervous system lymphoma (PCNSL), secondary central nervous system lymphoma (SCNSL)

## Abstract

Chimeric antigen receptor T-cell (CAR-T) therapy has transformed the management of relapsed/refractory B-cell lymphomas, yet its initial use in central nervous system lymphoma has been hampered by safety concerns and limited efficacy. Current data are derived from small, heterogeneous trials, with unresolved questions regarding neurotoxicity mitigation, optimal treatment timing, long-term response durability, and predictive biomarkers, precluding widespread clinical implementation. This narrative review systematically integrates peer-reviewed clinical trials, real-world retrospective datasets and preclinical mechanistic investigations to summarize the efficacy and safety profile of CAR-T in patients with PCNSL and SCNSL.

## 1 Introduction

Central nervous system lymphoma (CNSL), a rare subtype of non-Hodgkin’s lymphoma (NHL), is primarily classified into two primary CNSL (PCNSL) and secondary CNSL (SCNSL) (1). PCNSL has a much lower incidence than SCNSL and is highly aggressive, with unique clinical and biological features. It frequently affects the brain parenchyma, meninges, spinal cord, or eyes, without systemic dissemination. Notably, over 90% of reported PCNSL cases have been identified as diffuse large B-cell lymphomas (DLBCLs) (2). The prognosis for PCNSL is notably poor, with 5-year and 10-year survival rates reported at 29.9% and 22.2%, respectively. Furthermore, 10%–15% of patients undergoing methotrexate (MTX)-based combination therapy develop resistance to treatment and become refractory (3). The absence of a standardized and effective salvage therapy regimen for relapsed/refractory (R/R) PCNSL further exacerbates the unfavorable overall prognosis for these patients (4). In contrast, SCNSL is defined by secondary involvement of the neuroaxis resulting from systemic disease. Despite satisfactory therapeutic outcomes by implementing high-dose chemotherapy followed by autologous hematopoietic stem cell transplantation (HSCT), the prognosis remains suboptimal, with a pronounced risk of recurrence (5).

CNSL continues to pose significant clinical challenges owing to its poor prognosis and limited treatment options, especially in R/R cases (6, 7). The CNS exhibits a distinctive environment characterized by the blood-brain barrier (BBB), a vital structural barrier that can influence drug delivery and complicate disease management. Consequently, novel therapeutic strategies remain warranted to extend the duration of response (DoR) in patients with CNSL (5).

Recent advancements in genetic engineering have facilitated the development of CAR-T therapy, a prominent form of cancer immunotherapy. CAR-T has shown significant efficacy against hematologic malignancies, including B-acute lymphoblastic leukemia, DLBCL, multiple myeloma, mantle cell lymphoma, and follicular lymphoma (5, 8–11). The tumor microenvironment (TME) of PCNSL is rich in tumor-associated macrophages (TAMs) and tumor-infiltrating lymphocytes (TILs) (12). Although the unique TME of PCNSL and the presence of the BBB may influence the efficacy of CAR-T therapy, preclinical and clinical evidence support the effectiveness of CAR-T therapy in CNSL (13). Subsequent studies incorporating real-world data have also demonstrated the efficacy of CAR-T therapy in NHL with CNS involvement, showing a manageable safety profile (6). Notably, the majority of existing clinical datasets are limited by small sample sizes, heterogeneous patient populations, and single-arm study designs (14). Given persistent unmet clinical needs for R/R CNSL, this review critically integrates available clinical and translational data to characterize the safety and efficacy of CAR-T therapy in patients with primary and secondary CNSL.

## 2 Pathological mechanism of CNSL

The complex pathogenesis of CNSL involves molecular, cellular, and TME abnormalities. To date, several genetic variants associated with the development of CNSL have been identified (15, 16). Roschewski et al. (15) summarized the unique biological aspects of PCNSL: 1) The extremely high frequency of MYD88 (67-86%) and CD79B (61%) mutations suggests that impaired Toll-like receptor (TLR) and B cell receptor (BCR) signaling is a mechanism for enhancing NFκB activity; 2) Frequent mutations hinder tumor cells further differentiate and lock it in a GC-like proliferative state, including mutations in TBL1XR1, PRDM1, and translocations of BCL6; 3) the cell cycle negative regulator CDKN2A is almost universally deleted, with only rare mutations in TP53; 4) the frequency of mutations leading to immune evasion is very high, including deletions of HLA loci, or mutations in B2M or CD58. One study showed that B-cell lymphoma patients without TP53 mutations before conditioning showed better short-term efficacy of CAR-T therapy and were more likely to achieve CR. At the same time, the study showed that these 8 mutated genes were detected in 21 (30.88%) B-cell lymphoma patients: CDKN2A, CBLB, APC, SPEN, KMT2D, CARD11, FOXO1 and PDGFRB. The results indicate that patients without any of these mutations are more likely to achieve CR, and patients with any of these mutations have significantly lower OS and PFS, affecting the efficacy of CAR-T therapy (17). Given that MYD88L265P and CD79B are common in PCNSL and SCNSL subtypes, and that the Bruton’s tyrosine kinase (BTK) inhibitor ibrutinib inhibits chronically active BCR signaling and has selective activity against ABC-DLBCL tumors that also harbor this mutation, BTK is a highly plausible therapeutic target for CNS lymphoma (15).

At the cellular level, tumor cell growth patterns significantly contribute to the development of CNSL. Approximately 95% of PCNSL have exhibited a DLBCL pathological subtype, predominantly of non-germinal center origin. The remaining cases include marginal zone lymphoma (MZL), anaplastic large cell lymphoma (ALCL), Burkitt lymphoma (BL), lymphoblastic lymphoma (LBL), mature T/NKcell lymphoma (T/NK), and Hodgkin lymphoma (HL) (18). Indeed, single-cell sequencing results confirmed that PCNSLs consisted predominantly of tumorigenic B cells, which predominantly display activated B-cell-like phenotypes, including BCL6, CD10, and BCL2, while the remaining cells were mainly immune cells, oligodendrocytes, and fibroblasts. and tumor cells predominantly display activated (19). PCNSL tumor cells typically exhibit perivascular growth patterns accompanied by reactive perivascular T-cell infiltration, which constitutes their hallmark histopathological feature (20). SCNSL is distinguished from PCNSL as either synchronous CNS and systemic involvement of lymphoma or an isolated CNS relapse. SCNSL is not a specific biologic entity, but most cases are DLBCL (15).

In recent years, it has become increasingly evident that the biology of PCNSL is not solely determined by the tumor itself, but also by its close and complex interaction with the immune microenvironment. TAMs and dendritic cells, in particular, reshape the immune milieu in PCNSL (21). The tumor immunosuppressive microenvironment helps the tumor evade immune cell surveillance, thereby promoting tumor proliferation, infiltration, and metastasis (22). TILs express various immune checkpoints, including programmed cell death protein-1 (PD-1), cytotoxic T-lymphocyte antigen-4, T cell immunoglobulin and mucin domain-containing-3, lymphocyte activation gene-3, and T-cell immunoglobulin and ITIM domain (23). TAMs have been shown to express both PD-1 and programmed death-ligand 1 (PD-L1, CD274). Nina Worel et al. (24) found that when CAR-T are combined with PD-1 monoclonal antibodies in the treatment of relapsed/refractory diffuse large B-cell lymphoma, it can significantly improve the therapeutic effect of patients. And the frequency of differentiated CD3+CD27CD28- T cells predicts response to CAR-T therapy. TAMs interact with PCNSL cells, contributing to an immunosuppressive environment. Quantification of TAMs may have important prognostic implications (25). Therefore, anti-cancer therapies should target the tumor and aim to remodel the tumor microenvironment. And these immune checkpoints maintain immune tolerance and suppress autoimmunity.

In summary, the above findings suggest that targeted therapies and immunotherapy play an essential role in CNSL and may lead to significant breakthroughs in its treatment.

## 3 CAR T cell interaction with CNSL

CAR-T-based therapy has attracted significant attention in immunotherapy research for malignant tumors, representing a milestone breakthrough in targeted therapy (26). CAR-T therapy has yielded satisfactory outcomes in the treatment of various refractory or recurrent hematological malignancies and is considered a potentially curative approach for certain oncological conditions (27, 28). Studies have laid the foundation for the future development of CAR-T therapies for central nervous system tumors (29, 30).

Current therapeutic strategies for CNSL primarily include chemotherapy (31), immunotherapy (32), radiotherapy (33), and autologous stem cell transplantation (34, 35), each with inherent advantages and limitations. As a novel personalized immunotherapeutic approach, CAR-T therapy has shown distinct characteristics in the treatment of R/R CNSL compared to conventional therapies (36, 37) (**Table 1**). Available clinical data suggest CAR-T therapy can achieve high overall response rate (ORR) and CR rates with longer progression-free survival (PFS) among heavily pretreated R/R B-cell CNSL populations (38). However, its high cost, potential severe toxicity such as immune effector cell-associated neurotoxicity syndrome (ICANS), and antigen escape remain major challenges. In contrast, conventional therapies, such as High-Dose Chemotherapy (HDCT) and whole brain radiation therapy (WBRT), are more accessible but lack durable efficacy and are associated with significant long-term toxicity (39). The selection of therapeutic strategies should be individualized based on patient age, performance status, disease subtype, prior line of therapy, CNS lesion burden, and eligibility for CAR-T therapy. In contrast, conventional therapies remain the mainstay for frontline treatment or for patients ineligible for CAR-T therapy.

**Table 1:** Comparison of current therapeutic strategies and CAR-T therapy for central nervous system lymphoma (CNSL).

Therapeutic modality	Efficacy (R/R CNSL)	CNS penetration	Main toxicity	Advantages	Limitations
High-Dose Chemotherapy (HDCT) (31)	ORR: 40%-60%; CR:20%-30%; Median PFS: 4–6 months	Moderate; dependent on drug lipophilicity	Myelosuppression, neurotoxicity, organ damage	Widely available; relatively low cost	High relapse rate; poor tolerance in elderly/poor performance status (PS) patients
Monoclonal Antibodies (e.g., Rituximab, Polatuzumab Vedotin) (32)	ORR: 30%-50%; CR:15%-25%; Modest single-agent efficacy	Poor to moderate; limited by molecular weight	Infusion reactions, myelosuppression, infection	Low toxicity; combinable with chemotherapy	Limited monotherapy efficacy; drug resistance easily develops
Radiotherapy (Whole Brain Radiotherapy, WBRT) (33)	ORR: 50%-70%; CR:30%-40%; Short-term efficacy prominent	Excellent; covers all CNS lesions	Cognitive impairment, neurocognitive decline, myelosuppression	Rapid disease control; Suitable for bulky/extensive lesions	Long-term neurotoxicity; no durable remission; contraindicated in some elderly patients
Autologous Stem Cell Transplantation (ASCT) (34, 35)	ORR: 60%-70%; CR:40%-50%; Median PFS: 8–12 months	Indirect; relies on chemotherapy conditioning	Severe myelosuppression, infection, transplant-related mortality (TRM)	Potential for long-term remission; suitable for young/fit patients	Strict eligibility criteria; high toxicity; limited by prior therapy
CAR-T therapy (36, 37)	ORR: 60%-94%; CR:50%-67%; Median PFS: 7–13 months (CD19 CAR-T)	Good; engineered CAR-T can penetrate blood-brain barrier; ICV administration optional	ICANS, cytokine release syndrome (CRS), neurotoxicity	Durable remission potential; personalized; effective for R/R cases	High cost; limited availability; risk of severe toxicity; antigen escape

ORR, overall response rate; CR, complete remission; PFS, progression-free survival; ICANS, immune effector cell-associated neurotoxicity syndrome; CRS, cytokine release syndrome; PS, performance status; TRM, transplant-related mortality.

CAR-T therapy and CNSL exhibit complex interactions. CAR-T therapy for central nervous system lymphoma faces several challenges. The immunosuppressive microenvironment in the CNS may impair CAR-T function, limiting their anti-tumor efficacy. Prolonged exposure to modified cells also carries the risk of developing resistance to CAR-T therapy, particularly in patients with CD19 negative expression or CD19 splice variants not recognized by the scFv of CAR T cells in exon 2). Another cause of treatment resistance is loss of antigen or its downregulation (40). Additionally, this treatment may induce severe adverse reactions, such as cytokine release syndrome (CRS) and CAR-T cell-associated encephalopathy syndrome (41). However, these toxicities were manageable (42).

### 3.1 CAR T cells

**Figure 1:**
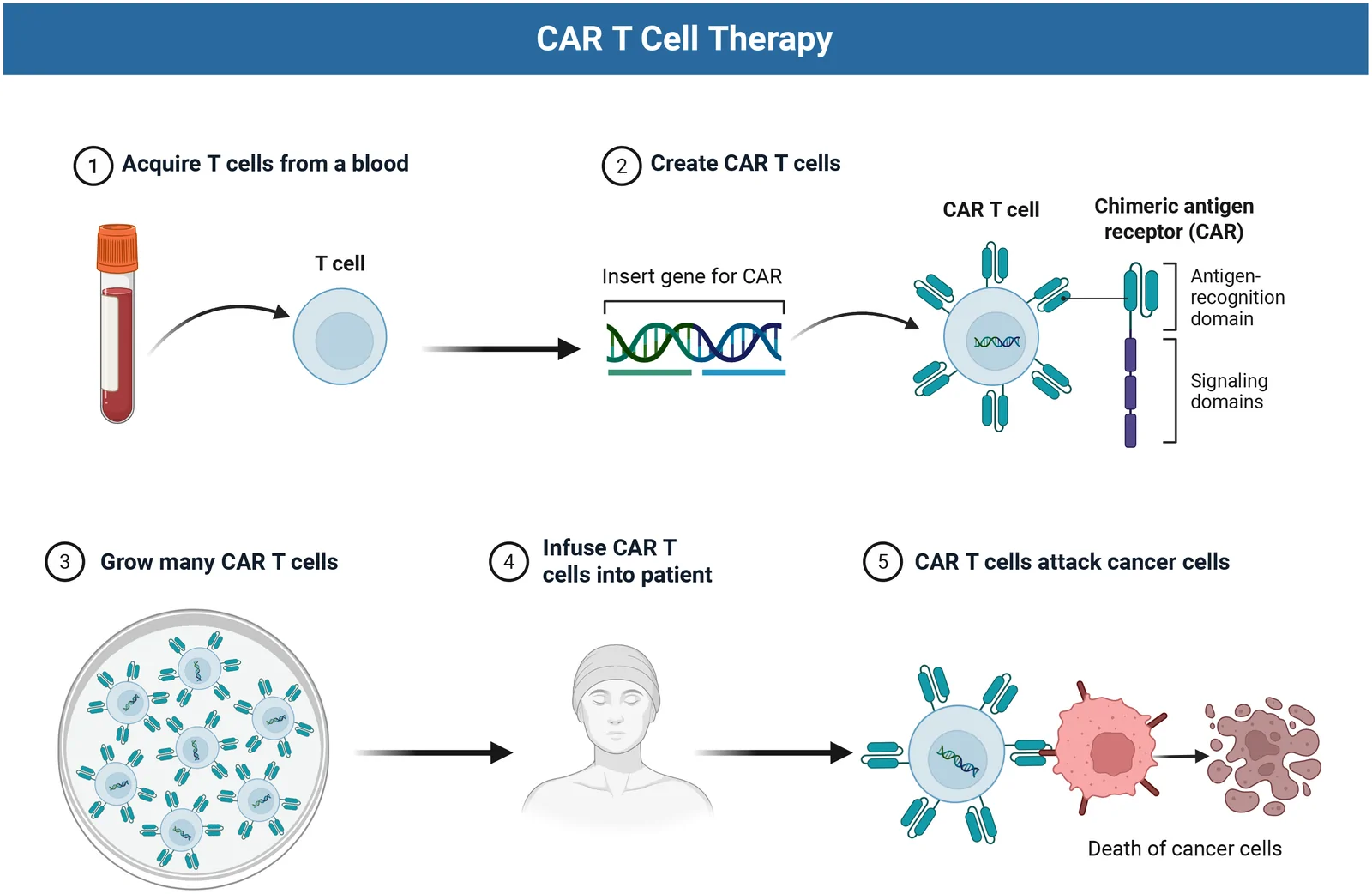
The process of CAR-T therapy. Peripheral blood samples are collected from patients. T cells are isolated and genetically engineered to present chimeric antigen receptors (CARs) and recognize a specific tumor associated antigen (TAAs). The obtained CAR T cells are expanded, and infused to the patient. CAR-T recognize and kill tumor cells. Created in BioRender.com.

CAR constructs consist of four modular core domains: antigen-recognition scFv, hinge, transmembrane region, and intracellular signaling motif. Upon tumor antigen binding, CD3ζ ITAM phosphorylation triggers downstream MAPK/NF-κB effector signaling, while costimulatory domains deliver secondary activation signals to support CAR-T proliferation, cytokine secretion, and tissue infiltration (13). CAR-T have undergone five generations of evolution (43). The first generation of CAR T cells was developed in 1993, consisting of a single-chain variable fragment (scFv) and an intracellular signaling domain (CD3) (44, 45). The second generation, an intracellular costimulatory domain (CD28 or 4-1BB) has been added in order to ameliorate T-cell proliferation and persistence, and has demonstrated considerable efficacy in treating hematologic malignancies (46–48). Third-generation CARs were obtained by including two different co-stimulatory domains, most commonly CD28 and 4-1BB, resulting in a “dual signaling” (49, 50). Fourth-generation CARs, termed TRUCKs (T cells redirected for universal cytokine killing), are designed to introduce additional transgenes to induce cytokine release. The intracellular signaling domain adds transgenic proteins such as IL-2 and cytokine costimulatory molecules such as CD27, and regulates the tumor microenvironment through the secretion of genetically modified cytokines (interleukins) (51, 52). Ultimately, in fifth generation, a binding site for STAT3 transcription factor and IL-2 receptor has been added to induce cytokine storm (43, 53). The full workflow of CAR-T manufacturing and clinical infusion is illustrated in **Figure 1**.

### 3.2 CAR-T therapy for CNSL

We separately analyze clinical evidence for PCNSL and SCNSL below, further stratifying data by study design (prospective phase I/II trial vs retrospective real-world cohort) and CAR product category (commercial single-target vs investigational dual-target CAR-T), to avoid purely descriptive narrative without critical comparison (**Table 2**). Multiple case reports, early-phase clinical trials and multi-center retrospective datasets confirm variable antitumor activity and divergent safety profiles of CAR-T against CNSL subtypes.

**Table 2:** Summary of study results of CAR-T for PCNSL/SCNSL..

Author	Year	Study design	Study subjects	CNS involvement (active vs historical)	Target/CAR-T products,	Response	Toxicity (CRS and ICANS at≥grade 3)	follow-up duration
Tu S. et al. (54)	2019	Case report	R/R PCNSL (n=1)	active n=1	CD19 and CD70	CR	--	17 months
Mizuta R. et al. (55)	2022	Case report	R/R PCNSL (n=1)	historical n=1	CD19 Tisa-cel	CR	--	13 months
Zou R et al. (4)	2023	Case report	R/R PCNSL (n=1)	active n=1	Tandem CD19/CD22 dual-targeted	CR	--	35 months
Li et al. (56)	2019	Phase I clinical trial	PCNSL (n=1) SCNSL (n=4)	active n=5	CD19 and CD22 in combination	at 60d CR =20%, PR=80%	ICANS G = 4(20%)	6–16 months
Wu et al. (57)	2021	Phase I	PCNSL = 4 SCNSL = 9 DLBCL	active n=2 historical n=11	ASCT + CD19 + CD22 (first line)	At 3m, ORR = 81.8% CR = 54.6%	ICANS G≥3 (7.6%)	14.2months
Siddiqi T et al. (58)	2021	Phase I	PCNSL (n=5) DLBCL	active n=5	CD19	At 28 d: CR = 60% SD = 40%	ICANS G = 3 (20.0%)	520 days
Frigault M.et al. (59)	2022	Phase 1/2	R/R PCNSL (n=12)	active n=12	Tisagenlecleucel	At 30 d CRR 50% ORR 58.3%	ICANS G≥3 8.3% (1/12)	12.2months (3.64 -23.5)
Nayak et al. (60)	2024	Phase I Abstract Session	PCNSL (n=13) SCNSL (n=4) DLBCL	active n=17	CD19 Axi-cel	At 3 month ORR = 94.0% CR = 67.0%	ICANS G≥3(28.0%)	24.2months
Alcantara et al. (14)	2022	Retrospective cohort	PCNSL (n=9) DLBCL	active n=9	Tisa-cel 7(77.8%) Axi-cel 2(22.2%)	At 30 d CR=33.3%, PR = 33.3% At 90 d CR=55.5%, PR = 11.1%	CRS G = 3 (14.3%) ICANS G≥3(28.6%)	8.5 months
Sylvain et al. (61)	2022	Retrospective cohor 64th ASH Annual Meeting Abstracts	PCNSL(n=17)	active n=17	CD19 (tisa-cel: n=13, axi-cel: n=4)	ORR = 76% CR = 52.9% PR = 23.5%	CRS G = 3 (5.9%) ICANS G≥3(17.6%)	17.8months
Choquet et al. (62)	2024	Retrospective cohort	PCNSL (n=25)	active n=25	Tisa-cel:n=16 Axi-cel:n=9	At 1 month ORR=76.0% CR = 32%	CRS G = 3 (8%) ICANS G≥3(20%)	20.8 months
Mercadal et al. (63)	2025	Retrospective cohort	PCNSL (n= 24)	active n=21 historical n=3	Tisa-cel 21 (88%) Axi-cel 3 (13%)	At 100 d ORR=61% CR= 48%	CRS G≥3(0%) ICANS G≥3(8.3%)	26months (3–38)
François Bouille (64)	2026	Letter to Editor Retrospective cohort	PCNSL (n=78) DLBCL	active n=78	Axi-cel n=46 Tisa-cel n=24 Liso-cel n=8	ORR=81% CR= 60%	CRS G≥3 (5%) ICANS G≥3(27%)	20.0months
Yu et al. (6)	2024	Retrospective cohort	PCNSL(n= 12) SCNSL (n= 10) DLBCL	active n=17 historical n=5	CD19 relmacabtagene autoleucel (relma-cel)	At 1m, ORR=90.9% CR = 68.2%	CRS G = 3 (4.5%) ICANS G≥3(4.5%)	316 days (55–618)
Lacan et al. (65)	2023	Retrospective cohort	PCNSL (n= 13) SCNSL (n= 8) DLBCL	active n=20 historical n=1	CD19 Tisa-cel (n=19) Axi-cel (n=2)	At 28d, ORR=67.0% CR = 29.0% PR = 38.0%	CRS G = 3 (4.7%) ICANS G≥3(9.5%)	12 months (1–29)
Cassanello et al. (66)	2025	Retrospective cohort	PCNSL (n= 8) SCNSL (n= 46) *De novo* LBCL = 34 Transformed NHL = 12	active n=39 historical n=10 Unknown n=5	Axi-cel n=16 Tisa-cel n=14 Liso-cel n=11 Sheba POC anti CD19 n=13	At 1month ORR=54% CR= 39% At 100d ORR=73% CR= 65%	CRS G≥3(11%) ICANS G≥3(28%)	(15.5–29.4) months
Anna Ossami Saidy1.et al. (67)	2025	Retrospective cohort	PCNSL (n= 16) SCNSL (n= 84) DLBCL 86 HGBCL (Myc +Bcl‐2± Bcl‐6) 6 Others^a^ 8	active n=67 historical n=33	Axi-cel n=59 Tisa-cel n=38 Liso-cel n=2 MB‐CART (investigational product, Miltenyi) n=1	At 180d ORR=92% CR= 41%	CRS G = 3(11%) ICANS G≥3(18%)	24.8months (19–29)
Shi et al. (7)	2025	Retrospective cohort	PCNSL (n= 19) SCNSL (n= 8) DLBCL n=5 PMBL n=1 BL n=1 Richter’s n=1	active n=27	CD19 n=24 CD20 n=2 CD19/CD22 n=1	At 1m ORR=88.9% CR= 85.2%	CRS G≥3(0%) ICANS G≥3(3.7%)	12 months
Frigault et al. (68)	2019	Phase I/II	SCNSL (n=8) DLBCL = 5 HGBCL = 2 PMBCL = 1	active n=8	CD19 Tisa-cel	ORR=58.3% CR = 50% PR = 16.7%	ICANS G≥3 (8.3%)	--
Abramson et al. (69)	2020	Phase I	SCNSL (n=7) DLBCL, HGBCL,PMBCL, tFLa	active n=7	CD19	CR = 50.0%	ICANS G = 3 (33.0%)	18.8months 15.0-19.3
He et al. (70)	2025	Phase 2 clinical trial	SCNSL (n= 21) B-ALL (11/21), NHL (10/21): DLBCL (7/21), Burkitt (1/21), HGBL (1/21), B-LBL (1/21)	active n=21	third-generation CAR (1928zT2).	At 3m ORR=57.1% B-ALL CR=67%(7/11) B-NHL CR=40%(4/10)	CRS G = 3(14%) ICANS G≥3(28.6%)	20.4months (1.0-36.3)
Bennani et al. (71)	2019	Retrospective cohort (abstract of American Society of Hematology)	SCNSL (n=17) (15 infused) DBCL = 76% (ABC DLBCL = 53%)	active n=5 historical n=10	CD19	At 1m, ORR = 59.0% At 3m, ORR = 31.0%	CRS G≥3(13.0%);ICANS G≥3 (33.0%)	10.1months 7.6-12.6
Ghafouri et al. (72)	2020	Retrospective cohort	SCNSL (n=5) PMBCL = 1 DLBCL = 1 tNHL = 3	active n=5	CD19 Axicabtagene	At 28 d: ORR = 80% CR = 60%	ICANS G≥3(40%)	155.0 days (86–208)
Ahmed et al. (73)	2021	Retrospective cohort	SCNSL (n=7) DLBCL	active n=6 historical n=1	CD19 Axi-cel Tisa-cel	CR = 85.7% PD = 14.3%	CRS G = 3 (14.3%) ICANS G≥3 (30%)	5.1months (1.6-7.2)
Karschnia et al. (74)	2022	Retrospective cohort	SCNSL(n=10) DLBCLn=6 trFL n=3 PTLD n=1	active n=10	CD19	At 30d, ORR=70.0% CR = 20.0%	CRS G = 3 (10%) ICANS G≥3(30%)	6 months (1–15)
Ayuk et al. (75)	2023	Retrospective cohort Research Letter	SCNSL (n=28) aDLBCL tFL;PMBCL;tMZL;tCLL,tHL;Richter;Burkitt	active n=28	CD19 Axi-cel n=14 Tisa-cel n=14	At 28d, ORR=64% CR = 32%	CRS G = 3 (21.0%) ICANS G≥3 (15.0%)	17months (10-25)
Epperla. et al. (76)	2023	Retrospective cohort	SCNSL (n=61) DLBCLn=50 trFL n=5 tMZL n=2 PMBLn=1 RT=1; PTLD=1; MCL = 1	active n=61	CD19 Axi-cel 30 Tisa-cel 19 Liso-cel 11 Brexu-cel 1	ORR=68.0% CR = 57.0%	CRS G≥3 (16.0%) ICANSG≥3(44.0%)	14.1months (11.7–23.5)
Alsouqi et al. (77)	2024	Retrospective cohort	SCNSL (n=113) DLBCL= 88 tFL= 13 HGBCL= 9 Burkitt Lymphoma =3	active n=86 historical n=27	Axi-cel44 (39%) Liso-cel23(20%) Tisa-cel46(41%)	At 1m, ORR=68% CR= 34%	CRS G≥3(3.3%) ICANS G≥3(32.3%)	10.7 months (6.5-30.6)

CR, complete remission; CRS, cytokine release syndrome; ICANS, immune effector cell-associated neurotoxicity syndrome; DLBCL, diffuse large B-cell lymphoma; R/R PCNSL, relapsed/refractory PCNSL; PD, progressed disease; PMBL Primary mediastinal large B-cell lymphoma;SCNSL, secondary central nervous system lymphoma;PTLD - posttransplant lymphoproliferative disorde.r^a^Three patients with PMBL, two patients with intravascular lymphoma, one patient each with gray zone lymphoma, intermediate DLBCL/Burkitt lymphoma, follicular lymphoma.

#### 3.2.1 CAR-T therapy R/R PCNSL: research advances

Advances in CAR-T therapy and improvements in CAR-T preparation techniques and design in recent years have enabled the inclusion of PCNSL patients in clinical trials of CAR-T therapy. CD19-targeted CAR-T cells are the most widely applied regimens for R/R PCNSL. Multiple single-center and multicenter investigations have reported encouraging clinical responses. An early case report verified that dual-targeted CAR-T against CD19 and CD70 induced complete remission (CR) without high-grade neurotoxicity in one R/R PCNSL patient (54). Decitabine-primed CD19/CD22 tandem CAR-T plus maintenance therapy sustained CR for 35 months (4), and single-agent tisagenlecleucel also achieved long-term remission in one relapsed patient (55). Case data carry publication bias and lack control groups, restricting generalizability.

Prospective phase I/II clinical trials targeting active PCNSL have mostly adopted commercially approved tisagenlecleucel CD19 CAR-T preparations with unified enrollment criteria. Several mixed PCNSL/SCNSL phase I trial combining CD19/CD22 tandem CAR-T plus ASCT achieved an 100% cohort ORR and 60% CR at 3 months, with 7.6% severe ICANS, though there were fewer patients in the PCNSL subgroup (56, 57). A small-sample phase I trial by Siddiqi et al. (58) enrolled five PCNSL patients with active intracranial disease, reporting a 60% 28-day CR rate and only a 20% incidence of grade ≥3 ICANS; the obvious limitation lies in the extremely limited sample size and insufficient long-term follow-up data beyond 12 months. Frigault et al. completed a standardized phase I/II trial including 12 active PCNSL patients who received tisagenlecleucel (59), showing a 58.3% ORR and 50% CR at the 30-day evaluation point, accompanied by low severe neurotoxicity at 8.3% grade ≥3 ICANS. Nayak et al. (60) reported at the meeting a phase I clinical trial involving PCNSL (n=13) and SCNSL (n=4) lymphoma. After treatment with CD19-Axi-cel, patients were evaluated for an ORR of 94.0% at 3 months, CR of 67.0%, ICANS ≥3 (28.0%), and a median follow-up time of 24.2 months.

Multicenter retrospective cohorts form the largest real-world evidence base. The French LOC cohort of 9 active PCNSL patients (14) saw CR rise from 33.3% at 30 days to 55.5% at 90 days, with 28.6% severe ICANS skewed by a high proportion of low-toxicity tisagenlecleucel. A retrospective study enrolling 17 PCNSL patients received CD19-targeted CAR-T reported an ORR of 76% (61). Medium-sized cohorts of 24–25 patients recorded 61%–76% 1-month ORR and 32%–48% CR, with rare severe CRS (0%–8%) and moderate ICANS (8.3%–20%). Extended follow-up (20.8–26 months) confirmed durable remission limited to early complete responders (62, 63). The 78-patient 2026 LOC registry (64) reported 81% ORR and 60% CR, with multifocal lesions and prior whole-brain radiotherapy identified as independent severe ICANS risk factors. Additional mixed-subgroup cohorts with CD19 CAR-T further validated broad ORR ranging 54%–92%,CRS≥3 (0–11%), ICANS≥3 (4.5–28%) (6, 7, 65–67).

Collectively, PCNSL data show commercial single CD19 CAR-T ORR 58.3% to 81% and CR 33.3% to 67%; dual/tandem CAR-T reached 81.8% to 89% ORR and outperformed single-target products for antigen-escape disease. Grade≥3 CRS remained uniformly low, while severe ICANS (3.7% to 68.2%) varied widely due to CAR-T costimulatory design, multifocal baseline lesions and prior cranial radiation. Nevertheless, the inconsistent response rates across studies and non-negligible severe ICANS still need to be addressed. Further large-scale prospective trials are required to standardize treatment regimens, optimize toxicity management.

#### 3.2.2 CAR-T therapy secondary CNS: research advances

SCNSL is another major subtype of CNSL, and CD19-directed CAR-T is also the mainstream treatment choice. Prospective phase I/II trials demonstrated short-term efficacy but elevated neurotoxic risk relative to PCNSL. Frigault et al. (68) treated 8 active SCNSL patients with tisagenlecleucel, attaining 58.3% ORR, 50% CR and only 8.3% severe ICANS. Small single-agent CD19 cohorts showed higher neurotoxicity: Abramson et al. (69) recorded 50% CR and 33% severe ICANS across 7 subjects. The phase II trial of third-generation dual-costimulatory CAR-T (70) produced 57.1% 3-month cohort ORR but merely 40% SCNSL-specific CR, accompanied by 28.6% grade ≥3 ICANS driven by amplified intracranial inflammation.CD19/CD22 tandem CAR-T induced partial remission in all 4 SCNSL patients with 20% grade 4 ICANS (56). Elevated systemic tumor burden and peripheral cytokine flux across a compromised BBB explain higher ICANS risk compared with PCNSL.

Retrospective registries reflected heterogeneous real-world outcomes with variable long-term durability. The 17-patient US cohort (71) exhibited marked efficacy decay: 59% 1-month ORR fell to 31% at 3 months alongside 33% severe ICANS, attributed to persistent systemic lymphoma reservoirs causing repeated CNS seeding. Ghafouri et al. (72) reported 80% ORR, 60% CR and 40% severe ICANS in 5 patients; Smaller cohorts reported 64%–70% 30-day ORR and low severe CRS (10%–15%) (73–75). Large international cohorts (66, 67, 76, 77) recorded 54%–68% 1-month ORR rising to 73%–92% at 90–180 days, with CR ranging 34%–65% and severe ICANS 18%–44%; multivariate analysis confirmed concurrent systemic extranodal disease as an independent neurotoxicity predictor. A Chinese real-world relma-cel cohort (6) achieved 90.9% 1-month ORR with minimal 4.5% severe ICANS, suggesting optimized lymphodepletion protocols may mitigate neurotoxicity; combined WBRT plus CAR-T also delivered favorable safety and efficacy (7).

Cross-subtype comparison reveals comparable short-term response rates between PCNSL and SCNSL, yet SCNSL carries higher relapse risk and shorter durable remission due to recurrent intracranial seeding from systemic disease. Median severe ICANS incidence in SCNSL cohorts (30%–44%) exceeds that of PCNSL (8%–28%), stemming from combined systemic and intracranial cytokine release. Dual-target CD19/CD22 CAR-T improves sustained CR rates for antigen-escape SCNSL, matching salvage performance observed in PCNSL mixed cohorts (55, 57). In conclusion, CAR-T therapy is an active and generally safe option for R/R CNSL, and CNS involvement should not preclude patients from receiving this treatment.

#### 3.2.3 Neurotoxicity of CAR-T therapy in CNSL

ICANS remains the primary safety limitation of CAR-T for CNS lymphoma, and published data show substantial inter-study heterogeneity in its incidence. The American Society for Transplantation and Cellular Therapy (ASTCT) grading is used in clinical practice to diagnose and grade CRS and ICANS. Regarding neurotoxicity, the results appeared reassuring in all the studies, with 3.7% to 44% of grade 3–4 ICANS, which is also close to what is reported in systemic DLBCL (78). ICANS arises acutely 3–7 days post-infusion with new-onset encephalopathy, aphasia or tremor; elevated CSF cytokines and CAR-T infiltration serve as specific biomarkers per ASTCT criteria (65). Prospective trials uniformly adopted the ASTCT 0–4 ICANS grading system (58, 59, 68, 70), while most retrospective cohorts used unstandardized institutional scoring. Active multifocal intracranial lesions double neurotoxic risk, whereas patients with only historical CNS relapse rarely develop severe ICANS. CAR-T design impacts toxicity: CD28-based axi-cel carries higher risk than 4-1BB-based products, and third-generation dual-costimulatory CARs further amplify intracranial inflammation (78). Prior whole-brain radiotherapy disrupts the blood-brain barrier and elevates the risk of severe ICANS (7). For SCNSL patients, concurrent systemic tumor burden triggers peripheral cytokine release that crosses compromised brain barriers, explaining higher median severe ICANS rates in SCNSL cohorts relative to PCNSL. Leptomeningeal involvement (with or without parenchymal involvement) portended a higher rate of ICANS in Epperla et al.’s series (76). Currently, there are limited available data on the management of neurotoxicity in CAR-T treatment of central nervous system lymphoma. Classic treatment with steroids is mainly used, and more studies are needed to provide a basis for further exploration.

### 3.3 Preclinical studies for CNSL

Preclinical studies have played a pivotal role in development of CAR-T therapy for treating CNSL. To overcome the physical obstacle of BBB to CAR-T therapy, the preclinical studies conducted by Mulazzani’s research team and Wang’s research team on *in situ* PCNSL mice used both intravenous and local intracerebroventricular injections, respectively. The results of both studies demonstrated that intracerebroventricularly infused CAR T cells had better anti-tumor effects. Furthermore, the CAR T cells exhibited superior proliferation and persistence, as well as a higher percentage of CAR T cells exhibiting a memory phenotype (79, 80). Furthermore, nanotechnology offers another avenue for treating CNSL. Nanocarriers can enhance drug delivery efficiency, facilitate CAR-T penetration across the BBB, and improve tumor-targeted accumulation (81). Ye et al. successfully constructed CAR macrophages (CAR-Ms) and CART cells using CARmRNA using lipid nanoparticles (LNPs). More importantly, both CAR macrophages and CART cells showed significant cytotoxic effects on B-cell lymphoma *in vitro (*82).

CAR T cells must surmount the immunosuppressive milieu when eradicating tumor cells; Therefore, remodeling the TME may effectively resolve this difficulty. Rodriguez Garcia et al. (83) discovered that FRβ-expressing TAMs have immunosuppressive M2-like features and that specific depletion of FRβ+ TAMs by CAR-T therapy can reprogram the TME *in vivo*, promote endogenous T-cell-mediated immune responses, control tumor progression, and synergize with CAR-T therapy targeting tumor antigens. Research shows that a biomimetic nanoformulation with chemokine “lever regulation” capabilities can establish matching migration signals to build an immune cell microenvironment that supports CAR-T therapy (84). These nanoformulations reshape the tumor chemokine microenvironment by upregulating immunogenic chemokines (CXCL9, CXCL10), thereby selectively recruiting CAR T cells; at the same time, they also act as nanobaits to neutralize CXCL12, thereby reducing the influx of immunosuppressive cells (such as CAR-Tregs and MDSCs), thereby enhancing the killing efficacy of CAR T cells.

Metabolic engineering of CAR T cells can be employed to better adapt them to the CNS microenvironment, enhancing their survival, proliferation capabilities and antitumor immunity (85, 86). Areej Akhtar et al. (87) developed metabolically reprogrammed CAR (MCAR) T cells with enhanced autophagy and mitophagy functions. A compound screen identified a synergistic effect between the GLP-1R agonist (semaglutide [SG]) and urolithin A (UrA) to maintain mitochondrial metabolism in CART cells (MCART-1). These changes enhance persistence and antitumor activity *in vitro* and in xenograft models. At the same time, GLP-1 secreting cells (MCART-2) MCART-2 cells can also reduce the risk of cytokine release syndrome (CRS). This strategy highlights the potential of metabolic reprogramming by targeting the autophagy/mitophagy pathway to improve CART cell therapy outcomes and ensure persistence and efficacy. Studies have found that CD8-positive T cells, macrophages, and chimeric antigen receptor (CAR) T cells expressing glucose transporter 5 (GLUT5) have enhanced effector functions in a glucose-limited environment *in vitro (*88).

## 4 Conclusion

**Figure 2:**
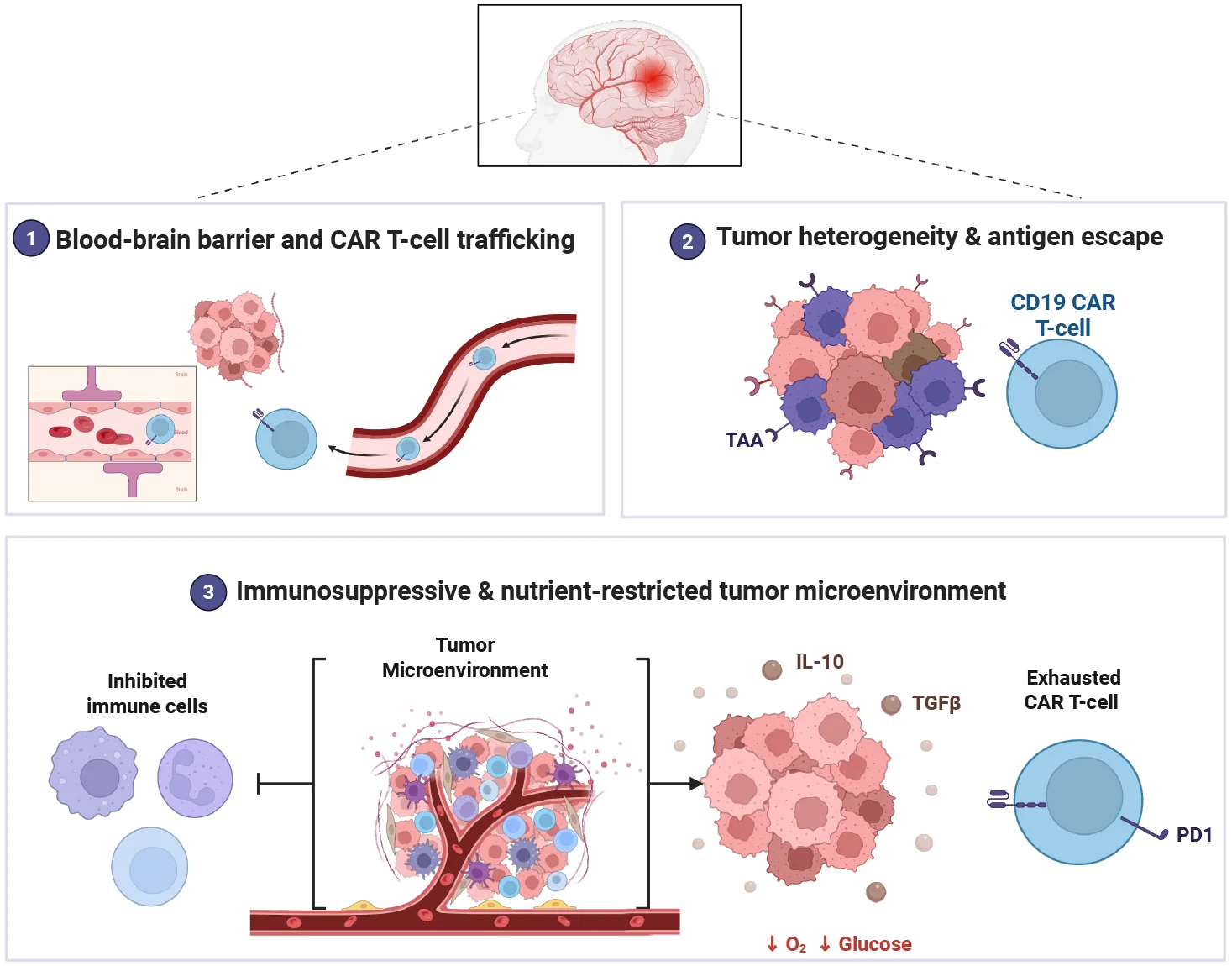
Challenges in CAR-T therapy for central nervous system lymphoma. (1) The blood-brain barrier restricts effective infiltration of CAR-T cells into tumor sites tumor-intrinsic resistance, and CAR-T trafficking in blood. (2) Tumor heterogeneity and antigen escape of CAR-T cells. (3) The immunosuppressive microenvironment in the central nervous system, including the presence of immunosuppressive cells and the secretion of immunosuppressive factors-suppresses CAR-T cell activity. Created in BioRender.com.

CAR-T therapy has demonstrated substantial potential for treating CNSL. However, numerous challenges remain to be addressed before clinical application (**Figure 2**). It cannot yet be regarded as standardized universal salvage treatment for all r/r PCNSL and SCNSL patients. As an emerging treatment modality, CAR-T therapy requires continued research to provide more reliable data and the development of appropriate methods and standardized processes to ensure the safety and effectiveness of the treatment. This approach aims to enhance efficacy, reduce side effects, and provide more effective treatment options for R/R CNSL, achieving long-term durable remission with CAR-T therapy.
